# Cardiac Shock Wave Therapy Improves Ventricular Function by Relieving Fibrosis Through PI3K/Akt Signaling Pathway: Evidence From a Rat Model of Post-infarction Heart Failure

**DOI:** 10.3389/fcvm.2021.693875

**Published:** 2021-06-16

**Authors:** Luqiao Wang, Xin Tian, Yuting Cao, Xuejuan Ma, Leilei Shang, Hao Li, Xueting Zhang, Furong Deng, Shumin Li, Tao Guo, Ping Yang

**Affiliations:** ^1^Department of Cardiology, The First Affiliated Hospital of Kunming Medical University, Kunming, China; ^2^Department of Cardiology, Suizhou Central Hospital, Suizhou, China; ^3^Department of Cardiology, Yunnan Fuwai Cardiovascular Hospital, Kunming, China

**Keywords:** cardiac shock wave therapy, heart failure, cardiac fibrosis, CD34/αSMA, PI3K/Akt signaling pathway

## Abstract

**Objection:** Cumulative studies have identified the effectiveness of cardiac shock wave therapy (CSWT) in treating heart failure after acute myocardial infarction (AMI), but little have been discussed with regard to the beneficial effects of CSWT on anti-fibrosis along with the underlying mechanism. In this study, we investigated whether CSWT could reduce post-AMI fibrosis and further explored the molecular mechanism.

**Methods:** Rat heart failure (HF) models induced by ligating the left anterior descending coronary artery were established and validated by echocardiography. Eligible animals were randomly categorized into five groups: the sham group, the HF group, the HF + CSWT group, the HF + LY294002 group, and the HF + CSWT + LY294002 group. The cardiac weight, serum level of BNP, NT-pro BNP and echocardiography parameters were measured to assess cardiac function in different groups. Masson's trichrome staining was used to assess the proportions of the fibrotic area. The expression level of CD34, αSMA was measured by RT-PCR, Immunohistochemistry and Immunofluorescent analyses and the level of PI3K/Akt was quantified by Immunohistochemistry and Western blotting.

**Results:** The application of CSWT significantly improved cardiac function and reduced myocardial fibrosis and level of CD34 and αSMA, compared to the HF group. CSWT led to significant elevations of p-PI3K and p-Akt expression levels compared to that of the HF group and the inhibition of the PI3K/Akt pathway abolished the observed beneficial effects of CSWT.

**Conclusion:** CSWT can facilitate the alleviation of cardiac fibrosis induced by AMI through the activation of PI3K/Akt signaling pathway.

## Introduction

Accounting for a staggering 30% of all deaths, ischemia heart disease (IHD) is the primary cause of global mortality and has long been considered as a life-threatening problem (1). The loss of normal cardiomyocytes initiated by myocardium infarction can eventually triggers the replacement of necrotic zone by fibrous scar tissue, which generally has invalid systolic function. This is known as ventricular remodeling and can finally result in post-infarction heart failure (HF). A greater increase in morbidity, mortality, and a poorer prognosis were observed among patients with HF following myocardial ischemia, due to the inextricable association of IHD and HF (2, 3). That existing therapies focused more on symptom improvement than ventricular remodeling avoidance has driven the desire for more curative strategies to reverse remodeled hearts in this aging society.

Serving as a dominating factor of the occurrence and development of post-infarction HF, cardiac fibrosis can be constantly seen in infarcted regions and border areas. The inhibition of fibrosis is gaining increasing attention for the reason that if effective anti-fibrosis can be achieved in infarcted myocardium and the border region, the pathophysiologic process of ventricular remodeling leading to HF may be restrained, and the prognosis of HF patients could be substantially improved. Therefore, novel therapies that focused on fibrosis antagonization should be considered as a therapeutic target for the management of IHD and related HF.

An emerging body of research has reported cardiac shock wave therapy (CSWT), an effective and non-invasive therapy mainly applied to ameliorate left ventricular remodeling after acute myocardial infarction (AMI) (4, 5). Our preceding work has demonstrated that hearts of rats with AMI treated by CSWT exhibited reductions of cardiomyocyte apoptosis index, shrinking fibrotic areas and satisfactory cardiac function parameters (6, 7). Involved mechanisms include the coronary micrangium arteriogenesis inducement, anti-apoptosis and anti-inflammation. Identically being one of the indispensable chains to post-infarction HF, however, AMI-induced cardiac fibrosis is surprisingly scanty in the field of CSWT. Two studies reported suppressed fibrotic extent by anti-inflammatory and decreasing the amount of fibrocytes in CSWT-treated hearts in animals (8, 9), nevertheless, it remains poorly-understood regarding the molecular mechanisms underlying these results.

In this study, we aimed to investigate the anti-fibrosis effect conferred by CSWT and further elucidate the mechanism of such benefit by building rat models of post-infarction HF.

## Materials and Methods

The animal protocol of this study was approved by the Institutional Animal Care and Use Committee (IACUC) of the Institutional Ethics Committee at the First Affiliated Hospital of Kunming Medical University (Yunnan, China) (Animal Ethics NO.Kmmu2021050). Operations and animal care performed in this study conformed to “Guide for the care and use of laboratory animals” (National Institutes of Health, volume 25, no. 28, revised 1996).

### Animal Models

Fifty adult male Sprague-Dawley (SD) rats with initial weight from 250 to 300 g were purchased from the Animal Laboratory of Kunming Medical University [Animal certification number: SYSK(Dian) 2005-0004]. All rats were inbred under a temperature-controlled environment with regular 12/12 light/dark cycles in cages and fed on commercial rat chow and water *ad libitum*. The rat model of heart failure was established as previously reported (10). Briefly, with chloral hydrate injected intra-peritoneally, animals were treated with left thoracotomy after endotracheal incubation and ventilator-assisted breathing (frequency: 75 breaths/min; inspiration/expiration: 1:1; tidal volume: 13 cc). An incision with pericardium exposure was inflicted on the fourth intercostal space and the chest was opened carefully. Followed by unfolding pericardium, the left anterior descending (LAD) occlusion was performed with 6-0 silk suture to induce acute myocardial infarction (AMI) model, which was identified by myocardium pathological change from reddish to blanching and AMI-specific ECG manifestations. Erythromycin ointment was then applied to local surgery wound post-operatively after the immediate close of the thoracic cavity by suturing, to avoid infections for 3 days.

### Echocardiography

Heart function assessment by Vivid E9 Color Doppler ultrasound system equipped with a 10.0-MHz 9L-D transducer (GE Inc., Vivid E9 system, Wingmed, Milwaukee, USA) was performed prior to and 4, 8 weeks after the surgery, as previously reported (11). Left ventricular end-systolic diameter (LVESD), left ventricular end-diastolic diameter (LVEDD), and left ventricular ejection fraction (LVEF) were directly measured via the long axial section of the left ventricular and averaged from 3 consecutive cardiac cycles, while fractional shortening (FS) was calculated by the equation as follows: FS = [(LVEDD – LVESD)/LVEDD] × 100%. The evaluation was conducted by an independent experimenter in an observer-blinded way.

### Animal Grouping and Treatment

Four weeks after the surgery, the surviving rats with heart failure proven by echocardiography (LVEF ranging from 35 to 50%) were randomly categorized into the following groups: the heart failure (HF) group (*n* = 9), the HF + cardiac shock wave therapy (CSWT) group (*n* = 9), the HF + LY294002 group (*n* = 9), and the HF + CSWT + LY294002 group (*n* = 9). Besides, nine rats underwent chest wall open surgery without LAD ligation were included as the sham group.

The procedure of CSWT treatment was depicted in detail in our previous works (6, 7). In short, inhaled isoflurane-anesthetized rats in the HF + CSWT and the HF + CSWT + LY294002 groups received a myocardium-focused shock wave, which was generated from the MODULITH SLC therapy device (Storz Medical, Lohstampfestr, Taegerwilen, Switzerland) with 200 impulses, 0.24 mJ/mm^2^ energy flux density and the frequency of 1 Hz. Initiated 4 weeks after AMI model establishment, the CSWT treatment was administrated three times a week lasting for 4 weeks. An identical anesthesia process was given in non-CSWT group animals.

As the special inhibitor of the PI3K/Akt signal pathway, LY294002 was used to investigate the role PI3K/Akt played in CSWT. Animals in HF + LY294002 and HF + CSWT + LY294002 groups were treated with an intraperitoneal injection of LY294002 with a dose of 100 mg/kg. The same volume of saline was given in groups without the inhibition of PI3K/Akt.

### Sample Collection

Whole blood was collected from rats' eye socket veins under peritoneal injection of 0.3 ml/100 g 10% chloral hydrate preoperatively, 4 weeks after the surgery and 4 weeks after the intervention. Experiment animals were executed 4 weeks after the intervention to obtain heart tissues. Before removing the hearts from the thoracic captivity, saline-based irrigation into LV was performed to purge the red blood cells. After that, the heart tissues were cut into cardiac apex and base, which were then fixed by 4% paraformaldehyde and preserved at the temperature of 4 and −80°C, respectively.

### BNP and NT-pro BNP Detection

Serum BNP and NT-pro BNP levels were quantified by Rat BNP and NT-pro BNP ELISA Kit (Yinghua Institute of Biotechnology, Beijing, China; Sabbiotech Inc., Shanghai, China) as instructed by the manufacturer. Serum samples were added to the wells and then incubated for 2 h, followed by 1-h incubation of responding antibody and 30-min incubation of streptavidin–peroxidase conjugate at room temperature. Afterward, the reaction was terminated by adding 50 μl stop solution after incubating chromogen substrate solution for 15 min. The concentration was confirmed by detecting the optical density (OD) of each well at 450 nm using a micro reader.

### Reverse Transcription Polymerase Chain Reaction

Rats left ventricular (LV) tissues were homogenized to obtain total RNA extractions by using TRIzol reagent (MRC Inc., Darmstadt, Germany), according to the producer's protocol. Following the directions of All-in-OneTM First-Strand-cDNA Synthesis Kit (GeneCopoeia Inc., Maryland, USA), 1 μl of RNA was used as a template for creating cDNA and real-time PCR was carried out on a type 2720 PCR instrument (Thermo Inc., Waltham, USA) in a final volume of 20 μl of reaction system that contained 10 μl SYBR Green/ROX qPCR Master Mix (GeneCopoeia Inc., Maryland, USA), 100 μg of cDNA, 0.16 μl of primer of each target RNA, and the corresponding volume of nuclease-free water with thermocycling conditions of 95°C for 10 min, 95°C for 15 s, 60°C for 20 s, and 72°C for 30 s, 40 cycles. The primer sequences were: GAPDH (forward) 5′-CAAGTTCAACGGCACAGTCAAGG-3′ and (reverse) 5′-ACATACTCAGCACCAGCATCACC-3′; CD34 (forward) 5′-TTCACAACCACAGACTTACCCAAC-3′ and (reverse) 5′-CCCTTTCCTTCTTAAACTCCTCAC-3′; αSMA (forward) 5′-ATCTGGAATCCCGAGTGACAAG-3′ ands (reverse) 5′-CGTGAAGAGGACCTGGGAGTAG-3′. The mRNA expression levels of CD34 and αSMA were determined by comparing that of the GAPDH, which was chosen as internal controls.

### Masson's Trichrome Staining

The Masson Staining Kit (Solarbio Inc., Beijing, China) was employed to observe the extent of myocardial fibrosis in five microscopic fields ( ×200) per 5-μm section of LV myocardium. The fibrosis percentage was defined as the ratio of fibrosis area and myocardial area, which was quantified via Image-pro Plus 6.0 (Media Cybernetics Inc., Bethesda, Maryland, USA).

### Immunohistochemical and Immunofluorescent Analyses

After de-waxing and hydrating, the prepared LV sections were initially incubated with 0.01 mmol/L citric acid solutions and then deionization water for 15 and 10 min, respectively, to inactivate endogenous peroxidase. For immunohistochemical analyses, serial sections were immunostained with primary antibodies against CD34, αSMA, and p-Akt (1:50, 1:100, 1:100, 1:200 rabbit polyclonal, respectively, both from Abcam Inc., Cambridge, Massachusetts, USA) at 4°C overnight and then incubated with the secondary biotinylated antibody for 20 min at 37°C. After diaminobenzidine (DAB) staining and hematoxylin counterstaining, tissue slices were subjected to regular dehydration, clearance, and cover. The proportion of positive cells for each slice were quantified by observing 10 randomly chosen microscopic fields ( ×400) and the averaged positive rates of five slices were identified as the final positive proportion for each target protein.

LV tissue sections were prepared as aforementioned. For endogenous peroxidase inactivation and membrane breaking, 0.01 mmol/L citric acid solutions and immunostaining permeate were used for 15 and 10 min, respectively. Thereafter, diluted primary antibodies against αSMA/CD34 (1:50/1:100, Abcam Inc., Cambridge, MA, USA) and Procollage-I/CD34 (1:100/1:100, Abcam Inc., Cambridge, MA, USA) were added to tissue sections for 4°C overnight incubation, followed by combined secondary antibodies for 40 min at 37°C (Beijing Ximeijie Technology Co., Ltd, Beijing, China). After PBS washing, Nuclei were stained with 1 mg/ml of DAPI (Boster Biotechnology Co. Ltd, Wuhan, China) for 7 min. The fluorescence analyses were performed with a confocal microscope (Olympus Tokyo, Japan).

### Statistical Analysis

All data were expressed as mean ± standard error of means (S.E.M) and analyzed by SPSS 19.0 (SPSS Inc., Chicago, IL, USA). Normal distribution and homogeneity test for a variance were conducted prior to One-way ANOVA analyses, which were used to compare differences between multiple groups. *P*-values < 0.05, two-tailed, were deemed statistically significant. Statistical charts were produced by using GraphPad Prism 6.0 (San Diego, CA, USA).

## Results

### Ischemic Heart Failure Model Establishment

The validity of the HF model is tested by transthoracic echocardiography and BNP, NT-pro BNP examination. Four weeks after the surgery, the ultrasound indexes revealed a considerable deterioration of cardiac function, when compared with baseline values (Baseline values: LVEDV 6.64 ± 0.44; LVESV 3.20 ± 0.45; FS% 51.70 ± 4.4; LVEF% 70.18 ± 2.30; 4 weeks later: LVEDV 7.95 ± 0.28; LVESV 5.76 ± 0.20; FS% 31.21 ± 3.21; LVEF% 30.42 ± 4.22). Meanwhile, heart weight, serum BNP, NT-pro BNP, the most wildly used HF indicators, were markedly elevated 4 weeks after the LAD ligation, suggesting a successful establishment of the HF model.

### Rats Heart Failure and Fibrosis Alleviation Effects of CSWT

#### CSWT Improved Cardiac Function of Rats With HF

**Figure 1:**
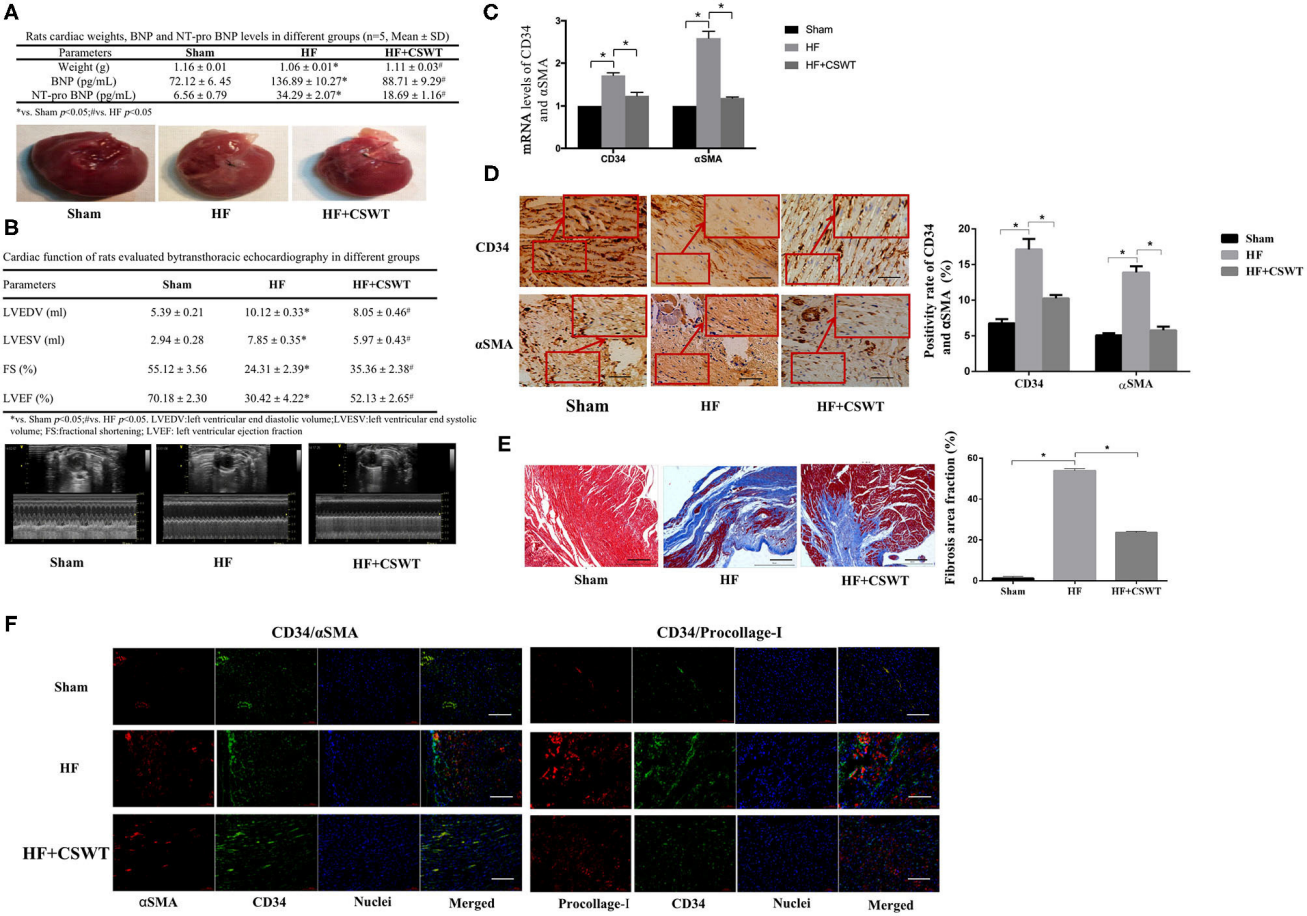
CSWT improves cardiac functions and reduced fibrosis in rats with post-AMI HF. **(A)** The cardiac weight, serum levels of BNP and NT-pro BNP and gross view of whole hearts in study groups; **(B)** Representative M-mode images by echocardiography of rats in study group; **(C)** Fold changes in CD34 and αSMA mRNA levels determined by RT-PCR in study groups; **(D)** The positive rates of CD34 and αSMA in study groups using Immunochemistry analyses; scale bar, 100 μm; **(E)** Representative images of Masson's trichrome staining and quantification for fibrosis of rat hearts from each group; scale bar, 100 μm; **(F)** Representative photomicrographs of immnunofluorescence for the detection of CD34/αSMA and CD34/Procollage-I. Red fluorescence shows αSMA or Procollage-I expression. Green fluorescence shows CD34 expression. Blue fluorescence shows nuclei of total cardiomyocytes; scale bar, 50 μm. Values are expressed as mean ± S.E.M (*n* = 5). One-way ANOVA test was applied for determining the significance of data. *p < 0.05. CSWT, cardiac shock wave therapy; AMI, acute myocardial infarction; HF, heart failure; S.E.M, standard error of means.

Rats' heart tissues were removed 8 weeks after AMI establishment. The general structure of hearts was shown in Figure 1A. Compared to the control, a less thickening regional wall of ventricular can be observed in the HF group while the CSWT group presented a thicker ventricular wall when comparing with that of the HF group. Quantitative analysis of heart weight suggested that hearts in the control group were heavier than that of in the HF group (1.16 ± 0.01 vs. 1.06 ± 0.01, *p* < 0.05) and heart weight in the HF + CSWT group were higher than that of in the HF group (1.11 ± 0.03 vs. 1.06 ± 0.01, *p* < 0.05). Besides, both the preoperative and post-operative serum levels of BNP, NT-pro BNP in 4 and 8 weeks were detected. As illustrated in Figure 1A, the results showed that BNP and NT-pro BNP had a lower level before the treatment than 4-week post-operative detection. Eight weeks following the surgery, in the HF group, the level of BNP and NT-pro BNP was markedly elevated compared to that of in the sham group. While it was decreased in rats receiving CSWT compared to the HF group.

Cardiac function was evaluated via transthoracic echocardiography, as presented in Figure 1B. Baseline cardiac function assessed before the treatment showed a relatively low tendency in LVESV and LVEDV and a high tendency in FS% and LVEF%, compared with the results obtained 4 weeks after LAD ligation. Furthermore, echocardiography parameters measured 8 weeks post-operatively exhibited significant deterioration of heart function in HF animals, as suggested by the disparity with the sham group (LVESV: 7.85 ± 0.35 vs. 2.94 ± 0.28, *p* < 0.05; LVEDV: 10.12 ± 0.33 vs. 5.39 ± 0.21, *p* < 0.05; FS%: 24.31 ± 2.39 vs. 55.12 ± 3.56, *p* < 0.05; LVEF%: 30.42 ± 4.22 vs. 70.18 ± 2.30, *p* < 0.05), whereas conspicuously improved in the CSWT + HF mouse, which yields markable discrepancies from HF mouse (LVESV: 5.97 ± 0.34 vs. 7.85 ± 0.35, *p* < 0.05; LVEDV: 8.05 ± 0.46 vs. 10.12 ± 0.33, *p* < 0.05; FS%: 35.36 ± 2.38 vs. 24.31 ± 2.39, *p* < 0.05; LVEF%: 52.13 ± 2.65 vs. 30.42 ± 4.22, *p* < 0.05).

#### CSWT Reduced Cardiac Fibrosis of Rats With HF

As Figure 1C showed, animal hearts were analyzed for CD34 and αSMA, which are two major fibrosis markers 4 weeks after CSWT via RT-PCR. As was revealed in Figure 1C, both CD34 and αSMA expression level increased in the condition of HF, compared with that of normal conditions (the HF group: CD34: 1.71 ± 0.06, *p* < 0.05; αSMA: 2.59 ± 0.16, *p* < 0.05), and the application of CSWT exhibited reversed tendency in comparison with that of the HF group (CD34: 1.24 ± 0.08 vs. 1.71 ± 0.06, *p* < 0.05; αSMA: 1.19 ± 0.01 vs. 2.59 ± 0.16, *p* < 0.05).

In addition, immunohistochemistry was used to assess the positive rate of CD34 and αSMA and the results showed that the positive expression rate of CD34 in myocardium tissues of the control and the HF group were 6.76 ± 0.58 and 17.13 ± 1.47% (*p* < 0.05), respectively (Figure 1D). However, the rate reached 10.25 ± 0.48% after applying CSWT, which was significantly heightened (*p* < 0.05). A similar trend was found in αSMA levels, which was strongly expressed in hearts from the HF group relative to the sham group (13.90 ± 0.85 vs. 5.07 ± 0.30%, *p* < 0.05), whereas was weakly expressed in the HF group with CSWT treatment, comparing to those in the HF group without CSWT administration (5.78 ± 0.51 vs. 5.07 ± 0.30%, *p* < 0.05).

Figure 1E presented the Masson's trichrome staining of rats LV myocardial sections from the sham, HF and HF + CSWT groups, in which the fibrosis region is blue-colored. It is suggested that, compared to the HF group, the collagen area proportion was obviously lower in the HF + CSWT group (the HF group: 53.89 ± 1.01, *p* < 0.05; the HF + CSWT group: 23.63 ± 0.54, *p* < 0.05).

Furthermore, the intensity of combinations of αSMA/CD34 and Procollage-I/CD34, two fibrotic indicators, were visualized by immunofluorescence staining (Figure 1F). The images showed higher levels of fibrosis in HF-suffered rats compared with those in untreated controls. Both two combinations were lightly-stained in CSWT-treated rats compared with that of the HF group.

### PI3K/Akt Signaling Pathway May Be Involved in the Effect of Anti-fibrosis

**Figure 2:**
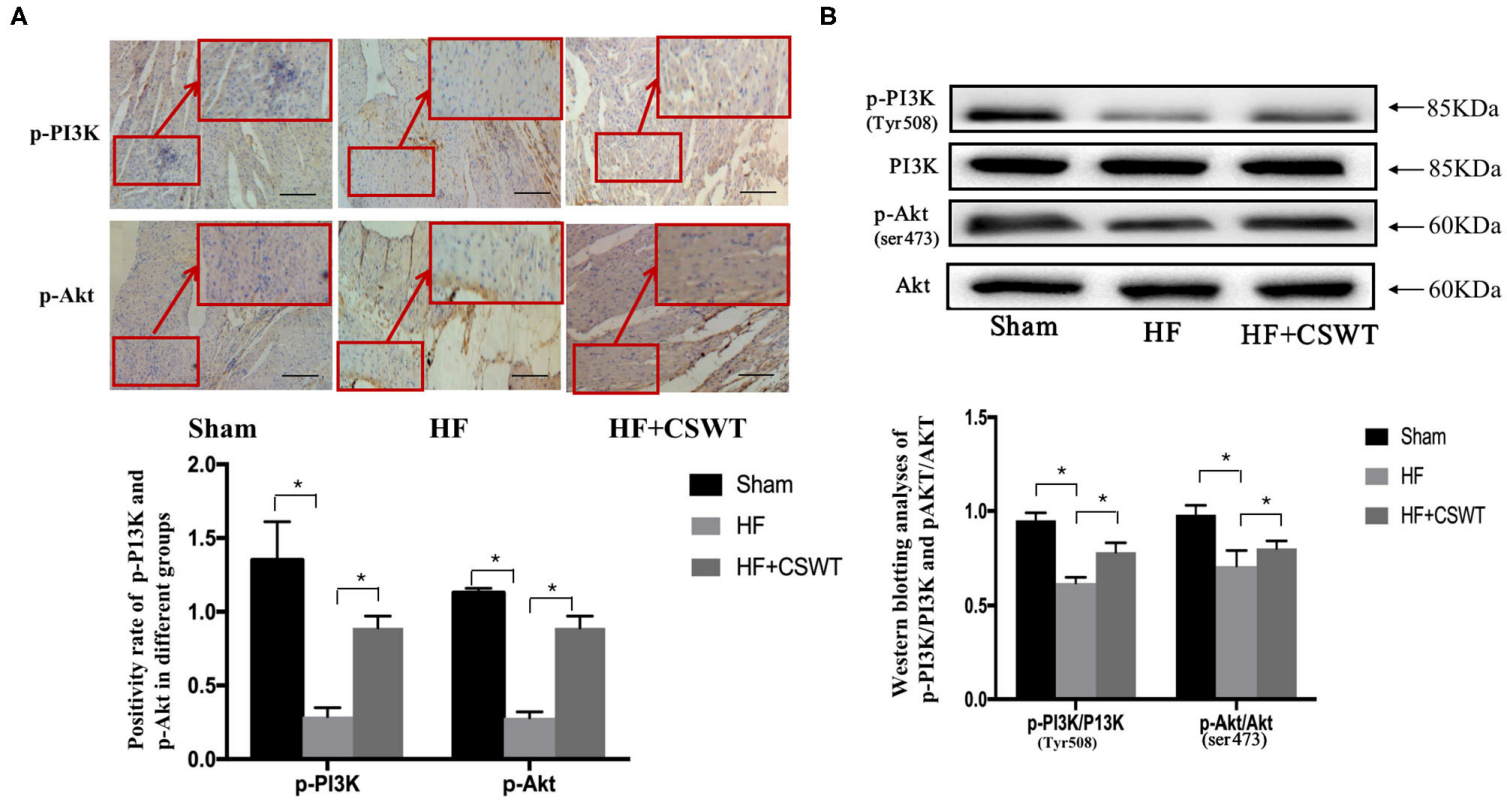
CSWT led to increased PI3K/AKT expression levels. **(A)** The positive rates of p-P13K and p-Akt in cardiocytes of rats in study group using Immunochemistry analyses; scale bar, 100 μm; **(B)** Relative protein expressions of PI3K, Akt, p-PI3K and p-Akt in cardiocytes of rats in study group using Western blot analyses. Values are expressed as mean ± S.E.M (*n* = 5). One-way ANOVA test was applied for determining the significance of data. *p < 0.05. CSWT, cardiac shock wave therapy; AMI, acute myocardial infarction; HF, heart failure; S.E.M, standard error of means.

Based on previous results that CSWT benefited cardiac function and alleviated myocardium fibrosis, we next examined whether PI3K/Akt signaling pathway, a key factor that has been shown to be responsible for withholding fibrotic progression after AMI, involved in the process of anti-fibrosis. Figure 2A presented specimens immunostained for p-PI3K and p-Akt. According to the analytic results from immunohistochemistry, the administration of CSWT led to a significant increase in the positive rates of p-PI3K and p-Akt, compared with that of the HF group. Meanwhile, as Figure 2B suggested, based on outcomes from western blotting analyses, the level of p-PI3K(Tyr508)/PI3K and p-Akt(ser473)/Akt were decreased in the HF group while CSWT treatment resulted in remarkable increase of p-PI3K(Tyr508) and p-Akt(ser473) levels.

### PI3K/Akt Pathway Mediated CSWT Effects of Anti-fibrosis

#### LY294002 Abolished the Cardiac Function Improvement Effects of CSWT

**Figure 3:**
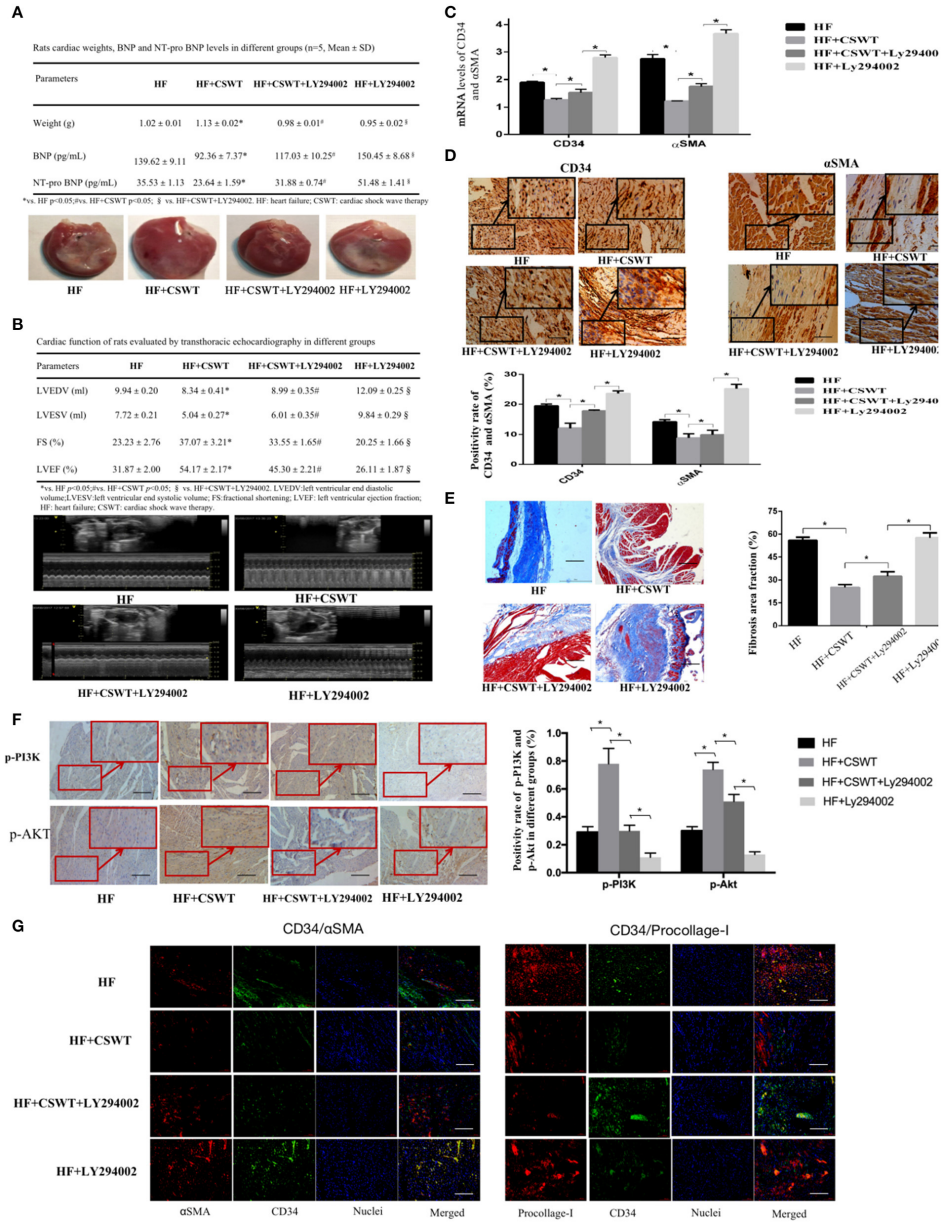
The inhibition of PI3K/Akt pathway abolished the cardiac function improvement and anti-fibrosis effects of CSWT. **(A)** The cardiac weight, serum levels of BNP and NT-pro BNP and gross view of whole hearts in study groups; **(B)** Representative M-mode images by echocardiography of rats in study group; **(C)** Fold changes in CD34 and αSMA mRNA levels determined by RT-PCR in study groups; **(D)** The positive rates of CD34 and αSMA in study groups using Immunochemistry analyses; scale bar, 100 μm; **(E)** Representative images of Masson's trichrome staining and quantification for fibrosis in cardiomyocytes of rats in study group; scale bar, 100 μm; **(F)** The positive rates of p-P13K and p-Akt in cardiomyocytes of rats in study group using Immunochemistry analyses; scale bar, 100 μm; **(G)** Representative photomicrographs of immnunofluorescence for the detection of CD34/αSMA and CD34/Procollage-I. Red fluorescence shows αSMA or Procollage-I expression. Green fluorescence shows CD34 expression. Blue fluorescence shows nuclei of total cardiomyocytes; scale bar, 50 μm. Values are expressed as mean ± S.E.M (*n* = 5). One-way ANOVA test was applied for determining the significance of data. *p < 0.05. CSWT, cardiac shock wave therapy; AMI, acute myocardial infarction; HF, heart failure; S.E.M, standard error of means.

Next, groups pretreated with LY294002 were set to inspect CSWT effects after PI3K/Akt pathway abolishment. Summarized cardiac weight of rats, level of BNP, NT-pro BNP along with the cardiac exteriors were listed in Figure 3A. Similarly, CSWT resulted in significantly higher cardiac weight, lower BNP, NT-pro BNP level. Nevertheless, P13K/Akt pathway inhibition eliminated such protective effects upon CSWT.

Post-AMI LV remodeling was assessed by LV dilatation, indicated by LVEDV and LVESV, and LV function, indicated by LVEF and FS. The representative images of echocardiography of animals in each group were exhibited in Figure 3B. In the HF + CSWT group, FS and LVEF were significantly improved compared with that of the HF group (FS: 37.07 ± 3.21 vs. 23.23 ± 2.76, *p* < 0.05; LVEF: 54.17 ± 2.17 vs. 31.87 ± 2.00, *p* < 0.05), while which were reduced in the HF + CSWT + LY294002 group (FS: 37.07 ± 3.21 vs. 33.55 ± 1.65, *p* < 0.05; LVEF: 54.17 ± 2.17 vs. 45.30 ± 2.21, *p* < 0.05). Similarly, LVEDV and LVESV were significantly enhanced in the HF group, which was significantly decreased in the HF + CSWT group (LVEDV: 9.94 ± 0.20 vs. 8.34 ± 0.41, *p* < 0.05; LVDSV: 7.72 ± 0.21 vs. 5.04 ± 0.27, *p* < 0.05). CSWT did not benefit FS, LVEF, LVEDV nor LVESV after inhibiting the PI3K/Akt signaling pathway, compared with animals in the HF + CSWT group.

#### LY294002 Abolished the Cardiac Anti-fibrosis Effects of CSWT

Lower mRNA level of CD34 and αSMA were noted in CSWT treatment group, however, which were again elevated after inactivating PI3K/Akt pathway, as shown in Figure 3C (CD34: the HF group:1.90 ± 0.14; the HF + CSWT group: 1.24 ± 0.08; the HF + CSWT + LY294002 group: 1.53 ± 0.12, HF + LY294002 2.79 ± 0.10, *p*-values < 0.05; αSMA: the HF group: 2.85 ± 0.16; the HF + CSWT group: 1.22 ± 0.01; the HF + CSWT + LY294002 group: 1.75 ± 0.10; the HF + LY294002 group: 3.67 ± 0.14, *p*-values < 0.05).

Meanwhile, the positive rates of CD34 and αSMA evaluated by immunohistochemistry showed the same pattern as mRNA levels among the four groups (Figure 3D). That is, the administration of CSWT led to fewer fibrosis-related molecular expressions and that the inhibition of PI3K/Akt caused variable increases in the positivity of CD34 and αSMA.

Myocardial sections subjected to Masson's trichrome staining were displayed in Figure 3E. As the results revealed, a significant reduction in the fibrotic area proportion was observed in the HF + CSWT group relative to the HF group, but this fraction was again elevated after adding LY294002 (the HF group: 53.89 ± 1.01%; the HF + CSWT group: 29.18 ± 1.73%; the HF + CSWT + LY294002 group: 32.25 ± 3.03%, the HF + LY294002 group: 57.63 ± 3.19, *p*-values < 0.05).

Immunohistochemical analyses of phosphorylated PI3K and Akt were shown in Figure 3F. The positive expression rates of both p-PI3K and p-Akt showed the lowest number in the HF + LY294002 group, indicating a successful establishment of the PI3K/Akt-inhibition model (p-PI3K: 0.29 ± 0.04; p-Akt: 0.13 ± 0.02). Moreover, the positivities of these two molecules showed a significant trend for elevation after CSWT administration, and then a trend for reduction after LY294002 pretreatment.

Figure 3G represented immunofluorescence examples of αSMA/CD34 and Procollage-I/CD34 combinations identified in heart tissues. The lowest fluorescence intensity of αSMA/CD34 and procollagen-I/CD34 were found in specimens from CSWT-treated rats. By comparison, both tissues from groups of the HF and the HF + LY294002 showed significantly increased levels of fibrosis markers, with the latter one being the highest.

Altogether, the before-mentioned outcomes convergingly supported our assumption that CSWT improved cardiac function in post-AMI HF by exerting anti-fibrosis effects through PI3K/Akt signaling pathway in rats.

## Discussion

When attacked by pathophysiological insults, two forms of cardiac fibrosis—replacement and reactive fibrosis would manifest as a response, and the former one plays a dominating role in the development of cardiac remodeling (12). It triggers the displacement of the myocardium with fibrous tissue, which delivers detrimental effects on the structure, excitation-contraction coupling, and the systolic and diastolic function of hearts. Therefore, cardiac fibrosis is recognized as a prerequisite for almost all forms of HF, predisposing patients with heart diseases to the outcome of HF (13). Also, it has been shown that fibrotic severity is directly and positively associated with the long-term mortality of HF patients (14). Based on our earlier work, CSWT has been found to result in satisfactory improvement of cardiomyocyte apoptosis both *in vitro* and *in vivo* (6). And our published paper showed that CSWT promoted arteriogenesis of coronary micrangium and alleviated fibrosis after AMI by integrin linked kinase (ILK)-induced inhibition of myocardial apoptosis (7). Another evidence was from Hiroaki's study (8). They found that extracorporeal low-energy shock-wave therapy significantly ameliorated LV remodeling and fibrosis in rat after acute AMI. The mechanisms may involved in suppressing the infiltration of neutrophils and macrophages and enhancing the expression of endothelial nitric oxide synthase. However, both our previous study and Hiroaki's study failed to fully observe the anti-fibrosis effect of CSWT, nor to explore its molecular mechanism and signaling pathways. Furthermore, the proven clinical benefits, together with the well-documented anti-fibrosis of CSWT hinted us that CSWT may take effect through suppressing the occurrence and development of fibrosis and thus serving as a promising procedure to improve the prognosis of patients living with HF. Nevertheless, what mediates the CSWT effect is scarcely discussed. To our best knowledge, the current study is among the first to fully evaluate the anti-fibrosis effect and further delve into the potential signal pathways underlying such effects of CSWT through multiple measurements from Echocardiography and Masson's trichrome staining to Immunohistochemical and immunofluorescent analyses.

Establishing a robust as well as stable HF model is the precondition for subsequent experiments. In this study, the measurements of LVEDV, LVESV, LVEF, and FS by echocardiography, the evaluations of the architecture, weight of hearts, and the detection of serum BNP, NT-pro BNP were conducted simultaneously 4 weeks after the surgery and CSWT application, which lend support to the reliability of our model. It is observed that, the cardiac volume of rats is enlarged 4 weeks after the LAD ligation, as indicated by the significantly heightened LVEDV and LVESV. The expanded LV volume stimulated the release of BNP, a cardiac function criterion that is directly proportional to the volume enlargement. Followed by the decrease of LVEF and FS, stemmed from the impaired myocardium function due to the injurious stimulus of ischemia and necrosis. In all, the above-mentioned data consistently suggested the fact that the current model meets our anticipation and was able to pave the way for our further experiments.

The fact that we investigated reduced CD34 and αSMA mRNA expression levels and positive rates, smaller fibrous areas, and lower density of fibrocytes in the HF + CSWT group than in the control group suggested that CSWT suppressed fibrosis-related molecules, such as CD34 and αSMA, leading to the improvement of cardiac fibrosis. Consistent with our findings, Lei et al. demonstrated that the role CSWT played in myocardial anti-fibrosis may be related to the repression of fibrocytes amounts in pigs (9). Similarly, the study of Abe et al., using a model of AMI rats, clarified that CSWT therapy attenuated cardiac fibrosis by reducing the number of TGF-β1-positive cells, an important signal that is well-acknowledged to be closely associated with the formation of fibrosis (8). The anti-fibrosis effect of CSWT is not limited to cardiac tissues, as a newly-published study has introduced this therapy in a model of liver fibrosis, which also obtained satisfactory outcomes (15). The potentials of CSWT were also supported by fibroblast originated from human hypertrophic scar (16). As a general process characterized by the accumulation of fibroblasts and abnormal deposition of the extracellular matrix, the development of cardiac fibrosis and liver or human hypertrophic scar had many similarities. Therefore, the current finding of the anti-fibrosis effect of CSWT is well-founded, however, it still needs to be further elucidated what types of collagen or procollagen were exactly the targets.

The PI3K/Akt signaling pathway is known as a regulator mainly responsible for cellular survival and functions (17). It has already been shown in our previous publication and unpublished data that this pathway was associated with the anti-apoptosis effect of CSWT both *in vivo* and *in vitro* (6). This finding concorded with the research of Yu et al., in which they reported that the PI3K/Akt pathway involved in suppressing apoptosis-related protein expressions and thus the apoptosis of cardiomyocytes by CSWT therapy in H9c2 cells (18). For a long time, apoptosis has been considered serving as an initiator or perpetrator in fibrotic response, and the activation of apoptosis can be observed in nearly all kinds of fibrosis. The potential mechanism involves immune modulation and paracrine signaling, which potentially and substantially contribute to the presence and persistence of fibrosis (19). The closely-bonded relation between apoptosis and fibrosis, together with the demonstrated mechanism of PI3K/Akt pathway underlying the cardiac apoptosis alleviation conferred by CSWT gave us a strong hint that this pathway may also explain the protective effect of CSWT in post-infarction fibrosis. Current findings are encouraging and supported our anticipation. By immunohistochemistry and western blot, it is found that phosphorylation of PI3K and Akt presented enhancive changes after undergoing CSWT while untreated ones showed opposite outcomes, indicating the potential involvement of PI3K/Akt in the beneficial effects of CSWT and also laying the ground for further experiments.

In the next step, we inhibited the PI3K/Akt pathway to find out whether the cardioprotective effects of CSWT were impaired or abolished. As we expected, blocking the PI3K/Akt pathway resulted in a reversed pattern, wherein the originally improved cardiac function, reduced fibrous areas and expressive amounts of CD34, αSMA, and Procollagen I showed opposite trends, compared with a single application of CSWT. These results implied that the cardioprotection of CSWT is achieved partially by ameliorating fibrosis after AMI and such effect may be mediated via a PI3K/Akt-dependent pathway. In recent years, there is growing evidence that the activation of the PI3K/Akt path facilitates the improvement of cardiac fibrosis in models from ischemia/reperfusion to AMI and diabetic cardiomyopathy (20–22). It's speculated that the phosphorylation of PI3K/Akt may help enhance the proliferation and suppress apoptosis and inflammatory responses via working with a series of related proteins. The proteins that have been discovered by now included vascular endothelial growth factor (VEGF) and nuclear factor-κB (23, 24). Additionally, in this study, despite the fact that LY294002 weakened the effects of CSWT on fibrosis, the CSWT-induced anti-fibrosis effect was not fully eliminated. It hinted us that there existed more pathways being functional in recovering AMI-induced cardiac fibrosis after applying CSWT. Therefore, future studies focusing on the synergy or antagonism with PI3K/Akt by other proteins or signaling pathways have to be discussed to gain a greater understanding of the working mode of CSWT.

This investigation offered novel insights into the molecular mechanism with regard to the anti-fibrosis benefits of CSWT. Nevertheless, it should be noted that the chemical inhibition of PI3K/Akt pathway rather than the PI3K/Akt genes knockout limits the strengths of current conclusion to determine the clinical application of CSWT. Despite of the preliminary character, this study proposed a new mechanism for the first time that the anti-fibrosis effect of CSWT may involved this signal pathway. To tremendously consolidate the current findings, future investigations with the PI3K/Akt genes knockout models is constantly needed.

## Conclusions

Taken together, CSWT is proved to promote the cardiac function indexes in rats with ischemic HF and deliver positive impacts on moderating cardiac fibrosis. Furthermore, we illuminated that the activation of the PI3K/Akt signal transduction pathway partially explained the biological effects that CSWT posed. These findings help expand the current understanding of CSWT-based HF therapy and it is certainly instigated that CSWT as an effective as well as non-invasive approach, is promised to protect hearts against fibrosis following ischemic HF.

## Data Availability Statement

The raw data supporting the conclusions of this article will be made available by the authors, without undue reservation.

## Ethics Statement

The animal study was reviewed and approved by the Institutional Animal Care and Use Committee (IACUC) of the Institutional Ethics Committee at the First Affiliated Hospital of Kunming Medical University (Yunnan, China).

## Author Contributions

PY designed and supervised the study. LW supervised the study and critically revised the draft. XT performed the statistical analyses and drafted the manuscript. XT, YC, XM, LS, HL, XZ, FD, SL, and TG performed the experiments. All authors contributed to the article and approved the submitted version.

## Conflict of Interest

The authors declare that the research was conducted in the absence of any commercial or financial relationships that could be construed as a potential conflict of interest.

## Abbreviations

CSWT: cardiac shock wave therapy
AMI: acute myocardial infarction
HF: heart failure
IHD: ischemia heart disease
IACUC: The Institutional Animal Care and Use Committee
SD: Sprague-Dawley
LAD: left anterior descending
LVESD: left ventricular end-systolic diameter
LVEDD: left ventricular end-diastolic diameter
LVEF: left ventricular ejection fraction
FS: fractional shortening
OD: optical density
LV: left ventricular
DAB: diaminobenzidine
S.E.M: standard error of means
VEGF: vascular endothelial growth factor.
